# Motivations of undergraduate student medical interpreters: Exposure and experience

**DOI:** 10.1186/s12909-024-05417-y

**Published:** 2024-04-24

**Authors:** Julie R. Wechsler, Susan Tamasi

**Affiliations:** 1 Emory University, Atlanta, GA USA; 2 University of Pennsylvania Perelman School of Medicine, Philadelphia, PA USA

**Keywords:** Medical interpretation, Undergraduate students, Interpreter training, Pre-health

## Abstract

**Background:**

When patients do not speak the same language as their doctors, they face poorer medical outcomes, decreased doctor-patient trust, and a diminished desire to seek medical care. It has been well established that interpretation is an essential part of an accessible healthcare system, but effective use of such language services relies on both the interpreters themselves and the healthcare teams working with them. This study presents an interdisciplinary examination of the motivations of undergraduate student medical interpreters, a group which serves as a bridge between these roles. While not full-time interpreters, they receive official training and spend time serving patients in local clinics. Further, for those who aspire to careers in medicine, interpreting provides invaluable exposure to the medical field and early professional know-how.

**Methods:**

Semi-structured individual interviews with undergraduate student interpreters were conducted to describe this multifaceted educational experience. A thematic analysis framework was employed to understand how and why they volunteer their time to interpret.

**Results:**

Motivations of student interpreters were found to fall under three general categories: (1) personal identity, or connection to family, language, and their career aspirations; (2) community engagement, or the opportunity to make a direct impact on patients at an early stage; and (3) pre-professional experience, both in general and specifically in healthcare. Each of these contributes to the view of a student medical interpreter as a unique contributor to language equity in medicine, as they provide language services in the short-term as well as set themselves up to be linguistically and culturally competent providers in the long-term.

**Conclusions:**

A greater understanding of student motivations adds to knowledge about language mediation and validates the utility of students in this role, encouraging the development of more student interpreter programs. Particularly in communities with high proportions of non-English speakers, these students can contribute to making medical care as inclusive and accessible as possible.

**Supplementary Information:**

The online version contains supplementary material available at 10.1186/s12909-024-05417-y.

## Background

Although most individual medical schools do not explicitly require English proficiency, one must have strong English language skills to become a doctor in the United States. Most institutions have English coursework as a prerequisite for admission; the Medical College Admissions Test (MCAT) and United States Medical Licensing Examinations (USMLE) are only offered in English; communication and writing skills are often explicitly required competencies for medical schools, with the implication that those assets are English-based; and schools generally only conduct their curricula in English. This trend also continues past medical school, since doctors who trained abroad must prove their English proficiency to be eligible to take the U.S. board certifying exams [1]. Because it is difficult for someone who does not speak English well to become a doctor in the U.S., English-speaking patients rarely struggle to find a language-concordant provider.

However, far from all patients speak English. According to a recent American Community Survey estimate, 8.4% of the U.S. population older than 5 years qualifies as Limited English Proficient (LEP), amounting to more than 25 million people who may have trouble navigating the English-speaking healthcare system [2]. This suggests that there must be supports in place to address the language barriers these patients face and provide equitable care.

Substantial previous research has shown that for non-English-speaking patients, interpreters markedly increase the caliber of healthcare interactions. Accuracy of diagnosis, patient understanding, adherence, patient satisfaction, and even some clinical markers all improve with an interpreter [3–5]. Many U.S. medical schools now include curricula on working with interpreters to ensure that providers do so effectively and achieve these outcomes [6]. There is, however, an opportunity for this learning to take place even earlier. For undergraduate students on pre-health career tracks, gaining an appreciation of the importance of interpretation could position students to become more sensitive to language barriers (and better prepared to deal with them) when the time comes to work with interpreters as providers themselves. With that in mind, here we consider what would motivate students to participate in such a role.

This study examines an undergraduate medical interpreter student organization at a major Southeastern research university as an example of a successful student interpretation program. In identifying why students are interested in interpretation, why they persevere through the lengthy training, and what they gain from it overall, we sought to understand the primary motivations of undergraduate students who choose to interpret.

### Present study

The program in this study, referred to hereafter by the pseudonym Student Volunteer Interpreters (SVI), is a chartered university organization composed of approximately 30 students from all class years. SVI accepts ten new students each year and partially subsidizes their participation in an official 40-hour training course led by professional interpreters. Students then coordinate volunteer interpreting sessions at local clinics for Spanish- and Portuguese-speaking patients. With several years of experience interpreting in a sizable metropolitan area, along with continued yearly recruitment of new interpreters, the group has shown a sustained commitment to serving Spanish and Portuguese speakers in their community.

Only a handful of programs that train students to be medical interpreters have been previously documented: Loyola University Chicago Stritch School of Medicine [7], Icahn School of Medicine at Mount Sinai [7, 8], Penn State College of Medicine [9], and Brown University [10]. Of these, most are for medical students, and only one other [10] includes both medical and undergraduate students. Other programs exist for college students to become certified interpreters, but these are mostly in non-healthcare settings (such as NGOs or community organizations [11]). SVI is thus unique in its focus on the medical setting and its inclusion of undergraduates.

All of these groups, including SVI, provide their students with dedicated interpreter education, albeit in different formats. Some design their own courses, while others use external companies’ materials. After passing internal application and interview stages, SVI students undergo an official 40-hour training course administered by an independent educational agency. The normally in-person training was converted to an online format in 2020 due to the COVID-19 pandemic, but this did not change the content; students still covered topics similar to those addressed in professional interpreter training programs such as the role of the interpreter, medical terminology and concepts, ethical considerations, access to language services, and preparation for certifying exams [12]. As mentioned, SVI has been able to reduce costs for students via grant funding, but the course itself costs upwards of $600 per student, making it a significant investment. Students in other programs reported that as a result of their training, they felt more comfortable interpreting, more easily grasped the purpose of an interpreter, and improved their medical vocabulary [8, 9]. With its unique combination of an official certification, dedicated training in a medical setting, and undergraduate participants, SVI merits closer study.

In the programs previously mentioned that are based in medical schools, it is obvious that students want to become healthcare providers. With SVI, college students are not yet bound to the field of medicine, yet they still commit substantial time, money, and effort to this activity. Therefore, this project aims to ask the following question: how does interpreting relate to students’ desire to go into medicine and their preparedness to do so? By evaluating reasons for interpreting, we examine one example of how undergraduates use extracurricular activities to make decisions about their career paths.

## Methods

### Participants

To explore the student interpreters’ motivations, one-time, semi-structured individual interviews were conducted with SVI members between September and December 2020. This study was evaluated by the Emory Institutional Review Board and determined to be exempt from further review.

As outsiders to this group, we obtained a list of current members from the SVI Executive Board and contacted those students individually via email about participating in the study. Seventeen responded with interest and participated in interviews. Each was compensated with a $10 gift card after the conclusion of data collection. Two spoke Portuguese and the rest spoke Spanish, and all self-described as having a very strong grasp of the language from having spoken their language at home throughout their lives (“fluent,” “native,” “proficient,” or the like). Some were born and currently reside in the U.S., others were born abroad and moved to the U.S., and the rest were international students who still lived elsewhere at the time of the interviews but came to the U.S. to attend college. The most noteworthy characteristic of this sample of interpreters was the unanimous intention to go into a career in healthcare. Table 1 lists relevant demographic characteristics of the interviewees.

**Table 1:** Demographic characteristics of SVI interviewees

Demographic characteristics	Number of students (%)
Age	Avg. = 19.4 (range: 18–21)
Gender	Female: 12 (70.5%) Male: 5 (29.4%)
Ethnicity*	Hispanic: 4 (23.5%) Hispanic/Latinx: 7 (41.2%) Latinx: 4 (23.5%) Mexican: 1 (5.9%) Did not respond: 1 (5.9%)
Year in school	First-year: 2 (11.8%) Sophomore: 6 (35.2%) Junior: 5 (29.4%) Senior: 3 (17.6%) Post-graduate: 1 (5.9%)
Places where students live or have lived	Brazil, Cuba, Ecuador, El Salvador, Honduras, Mexico, Venezuela
Career interests	Medicine: 8 (47.1%) Medicine + other (business, public health): 4 (23.5%) Nursing: 2 (11.8%) Dentistry: 1 (5.9%) Undecided healthcare: 2 (11.8%)

*Students were asked about their ethnicity through open-ended discussions, which led to variability in responses.

Because the yearly application process for SVI occurs early in the fall semester, seven participants were new interpreters completing the training at the time of the interviews. None of these new interpreters had yet had the opportunity to interpret in clinics. The remaining ten were at least second-year students who had previously completed the fall training course and had interpreted with SVI for at least one full semester. These students ranged in experience; some had interpreted several times over the course of their time in SVI, while others were new interpreters during the 2019–2020 school year. The latter group may have only interpreted once in person (or not at all) before the COVID-19 pandemic caused all in-person interpretation sessions to be canceled in Spring and Fall 2020. The resulting shift to on-call remote interpretation meant that interpretation sessions were more intermittent for all members of the group. Despite differences in amount of experience, these ten students were assessed as a single group of “more experienced” interpreters because they had comparatively more interpreting exposure than the students going through training. Importantly, all the interviewees were committed members of SVI at the time of the interviews and had completed or were in the process of completing the required 40-hour training course.

### Interviews

Interviews were conducted in English and followed a semi-structured format, starting with an introduction to the study, informed verbal consent, demographic information, and language background. The bulk of the interview then focused on participants’ initial reasons for joining and a description of that process (e.g. *When did you first get involved in SVI?*); how the interpretation sessions typically proceed, including specific memorable examples (e.g. *Describe a typical encounter with a patient and doctor*); a discussion of the benefits and challenges they face while interpreting; what their role entails (e.g. *How do you see yourself in comparison to professional interpreters?*); information about being a member of SVI (e.g. *Please describe your interactions with the other student members of SVI*); as well as a request for their advice to other student interpreters. The interview guide was adjusted slightly for newer interpreters to accommodate for differences between the groups (see the Appendix for the full interview guide; contact the corresponding author for the full interview codebook).

Each interview was conducted and recorded over Zoom and lasted 30–60 min (average: 36 min). *Otter.ai* software, integrated into Zoom, was used to auto-transcribe the recordings, then raw transcripts were checked against the video recordings to correct minor errors and ensure transcription accuracy. After the interviews, evaluation was guided by a thematic analysis approach, as laid out originally by Braun and Clarke [13] and later described by Kiger and Varpio [14]. Because the data contained rich examples and explanations, this method of finding salient themes from a set of qualitative data was an ideal framework for pulling trends and commonalities out of the corpus. Reliability of the resulting codebook was checked by having two researchers (one unaffiliated with the project) independently code a portion of the data based on the established codebook and check for agreement, such that a total of approximately 10% (by number of words) of the overall interview data was examined. The final round of coding showed strong agreement, defined as a consensus in the selection and location of codes for nearly all of the data by the second round.

## Results and discussion

The way people think, act, and make decisions as pre-health students has implications for how they will think, act, and make decisions later in their medical education [22]. Through the examples they shared, SVI students highlight the impact interpreting has had on their aspirations and preparation for medical careers thus far; from this, we can also make predictions for how interpreting might influence those future careers. The interviews demonstrated three broad categories of themes: (1) the role of the interpreter, (2) potential barriers to interpretation, and (3) motivations for interpretation. Here, we focus on pre-health motivations, a subset of the final category, as this is most relevant to the students’ identities as pre-health students, and because barriers and role perceptions have been outlined in previous scholarship [10, 15–17]. Such motivations are multilayered, but a critical component was the distinction between both *exposure to* medicine, which is a more passive activity that fosters a desire to enter the field, and *experience in* the medical field, a more active pursuit that prepares them to participate in medical care. In this section, we present analysis and quotations describing how both facets of interpretation enhance student development—they are helpful to students personally, build skills, help the community, and form a foundation for future practice.

### Exposure to medicine

Though perhaps somewhat obvious, it is worth stating that *medical* interpretation prepares students who aspire to careers in medicine. Whether students plan to go to medical school, nursing school, or other avenues of study, working directly with physicians and advanced practice providers affords them insight into, skills for, and personal connections to their futures in healthcare. An important and commonly cited motivation was that interpretation provides this kind of unique exposure to the medical setting. Students witness medical encounters firsthand, catering to their intrinsic interest in the field. More than simply observing, however, they actually participate, learning what it feels like to take an active role in this setting. By both watching and doing, they gain tools for their professional futures. SVI students derive great value from the chance to familiarize themselves with this setting:

“Me personally, I get a lot of experience from it. Especially for volunteering hours, I know it’s a résumé booster, but it’s also just a really good way to see what a clinic setting is like, what a hospital setting is like.” (I2^1^).

“I refer to it as a free sample, kind of, of the career I’m interested in. […] It made me aware of how this whole process actually works, and not just looking at it from the outside.” (I7).

For students who have had little experience with healthcare in the past, interpretation is a chance to step into the field. In this way, it overlaps with medical volunteering and particularly with shadowing, which generally involves a student observing a doctor’s daily practice. Shadowing helps students learn more about what careers they may be best suited for, make network connections, and gain mentorship from people already involved in their area of interest [18]. But because it is mainly intended for the student to make career decisions, it can be argued that it is not in the patient’s best interest [19]. Medical interpreting is perhaps more of a middle ground; since the interpreter’s presence is intended to improve the patient’s ability to communicate and, by extension, overall experience, its impact on patients may add more value than shadowing alone. It also prepares students to approach visits with humility, because having to interpret bidirectionally not only teaches the provider’s perspective but reminds them that the patient’s perspective holds equal weight.

Whether students see it as akin to shadowing or not, the blend of observation and action that comprises language interpretation is personally relevant and interesting to those going into healthcare, and was one of the strongest observed motivators for SVI students; all but two interpreters described the healthcare experience as a primary draw to interpreting.

### Experience in the medical field

While visualizing themselves in the role of a healthcare provider is useful, it is the interpreter’s direct participation that sets interpretation apart from other activities. Interpreters mentioned that the ability to have a tangible impact on patient experiences, especially at such an early stage, was a strong motivator:

“It’s really fun to just go to the clinics and it really attracted me, this knowing that I was going to be interacting with patients when if I were not in this club, I wouldn’t probably be interacting with patients until I got into med school.” (I4).

“I think it was really great to be exposed to medicine like that, to be a player in that interaction, not just observing. I think that was a big difference in other types of exposures to health care that I had had before.” (I9).

In addition to the personal gratification that comes with helping people directly, students’ work as interpreters helps fill a community need. The Association of American Medical Colleges (AAMC) places “service orientation” first on their list of important competencies, underlining this as an important characteristic of future medical professionals [20, 21]. Interpreting is a strong indicator of a student’s dedication to service—it demonstrates sustained commitment, an awareness of social determinants of health, and a wish to improve lives within the interpreters’ own community. Choosing to be involved in this at an early stage of training might foreshadow the intention to be community-oriented providers in the future.

On the other hand, pre-health students often aim to accumulate experiences that demonstrate their commitment to the field. While some experiences may be truly intrinsically motivated, others are valued more for their utility in getting into professional school [22]. Very few SVI students even mentioned their résumés and almost none talked about medical school applications, but it is possible that some interpret because it bolsters their résumés, though they choose not to identify this as a main motivator. However, when résumés did come up, students explained that they value the certification as a signal to others that they can provide linguistic services. This suggests that there may be both intrinsic motivation and acknowledgement of the benefit that comes with checking a box to show that they have performed community service.

Regardless of whether a student’s primary motivation is to get into a health professional school, participation involves significant commitment, and the hands-on nature of this involvement teaches skills relevant to clinical practice. Students described communication skills, general professional skills, and dedication to a difficult task as important competencies they take away from interpreting:

“[Interpretation] works on so many different professional skills like learning how to look at someone in the eye, and learning how to give hard truths that you have to say sometimes, and just learning how to say it in a way that doesn’t come off with any bias.” (I3).

“For me, I think [the benefit of interpretation is] definitely developing better communication skills and getting rid of my shy side, and just [being] able to also develop professional ethics and… yeah, a professional side.” (I13).

These qualities, along with others mentioned like improvement in leadership and language abilities, are applicable to plenty of professional scenarios, such as interacting with senior colleagues, receiving feedback from a supervisor, giving presentations, or speaking with people outside of one’s normal working group. Learning these strategies early on gives students a glimpse of how to deal with future professional interactions and challenges. Observing them in a clinic specifically may also be a helpful step toward a future of effective work and problem-solving in a medical setting.

As providers, students will also need to employ interpersonal skills to connect with patients, with empathy being an important one previously described in the literature [23, 24]. The extent to which so-called “soft skills” can be taught is debated [25–27], but SVI students in this sample did appear to draw a connection between such “soft skills” and interpreting. Six interpreters specifically mentioned empathy, and several others, through their descriptions of how they interacted with patients, showed a similar emphasis on visualizing themselves in the patient’s situation. Through the use of language like “imagine how difficult it is” (I5, I9, I14) or “I know how hard it is” (I16) to be in that situation, interpreters can picture themselves in the position of not being able to speak English in an important context like the medical setting, even if they have not experienced that personally (though several mentioned that they or their loved ones had indeed experienced this). When students discuss empathy in health professional schools—an increasingly common topic in these curricula—they learn some of the same tools that interpretation fosters, like eye contact and active listening [28]. Having practiced these skills early on, SVI students may be poised to be more prepared for creating a positive interpersonal environment in their future exam rooms.

SVI sets students up with general skills that they can employ in practice anywhere, but the fact that this group provides Spanish and Portuguese interpretation hints at the particular communities that students may want to serve in the future. Students clearly recognized that through interpreting, they are taking an active role in their immediate community—a community with which nearly all of them identify personally—to lower language barriers. They are not just interpreting for their own interest in medicine, or to do broadly defined community service, but because they are uniquely equipped to step up and serve in this way:

“I had never really used being bilingual in any way, only with my own life and my family, but I had never really used it to impact anything. So I thought [SVI] was really interesting and I wanted to get involved. […] It felt so liberating to be able to use it and make an impact.” (I10).

“If you’re capable to dedicate your time to helping and are lucky enough to have grown up with another language, I think it’s really important to use it for […] something bigger than yourself and contribute back to your community. […] If you can help with the language barrier, that’s one thing out of so many disparities that you can contribute towards easing.” (I14).

Unlike with other volunteer roles, SVI students have an opportunity to use their linguistic and cultural identities as tools that allow them to contribute to language equity. Interviewees referenced personal linguistic and family histories, mentioning that they “imagine everyone as like my family” (I16) or want to help the community “as if they were like my family” (I8). This notion of family ties suggests a direct connection between interpreters and patients that anchors the students into the community quite deeply. Having experienced such a strong desire to serve patients may have important implications for how they will practice medicine later.

One final—and important—point on the topic of professional development was students’ impressions of what it would mean to be a bilingual physician. Considering that SVI students have sufficient language proficiency to interpret, they will likely not need an interpreter for patients who speak Spanish or Portuguese when they are practicing healthcare providers. Several mentioned that they look forward to becoming bilingual providers:

“When I’m a nurse practitioner, I’ll have that experience, not only from school about what these diseases are, what the medical terminology is, but also I’ll actually be able to talk to my patient one-on-one without the need of somebody there because I’ve already interpreted before, so I already know how to speak to them directly. So I can actually form that connection with them. And I think that’s very helpful.” (I13).

“I want to be a bilingual doctor. I want to be able to have a patient, and if they don’t speak any English, I don’t want them to need an interpreter. I want to be the person that’s able to do it on my own and I think that this is really a big step towards reaching that goal because I’m learning medical terminology and I’m only planning on learning more from here. And also it would allow me to be a provider that has a better connection with my patients. Just being able to understand or relate or connect with them through a cultural or linguistic level other than English means a lot to patients. Especially growing up, I know my mom was like, ‘I like this doctor because they speak Spanish.’ You know what I mean? So it helps you build a better connection with the people that you are serving.” (I14).

These students demonstrate an awareness of how interpretation prepares them to become bilingual providers. Since it has been shown that patients are more likely to be satisfied when they speak the same language as their providers, having smoother and more direct communication may lead to improved patient satisfaction [29].

Most of the students in this group talked about speaking their own language in their future practice, but it seems that their experience as interpreters is perhaps the best preparation for working with interpreters for other languages as well. With a clear understanding of the role of the interpreter as part of the medical team, as well as some of the challenges that it entails, we would predict that they will be better able to facilitate interpreted visits from the provider side.

## Conclusion

This study examined the motivations for participation in SVI, a student interpreter program at a major Southeastern research university. Interviews revealed that SVI students are motivated by personal connection through language and culture, the opportunity to positively contribute to their communities, and pre-professional preparation. The lessons they learn through interpreting—intangible communication skills, more concrete awareness of language barriers, and improved capacity in their own languages—have important implications for their future careers. With this unique exposure to the medical setting, students are building tools to care for LEP patients who happen to share their own language, as well as any patient regardless of language. In this way, the impact of student interpretation is two-fold. First, students benefit from early and hands-on exposure to the medical setting, fostering both their interest and related key skills. Second, patients and the healthcare system could benefit in the long run if interpretation effectively trains future professionals to be better equipped to deal with language barriers.

Although we were able to obtain a good deal of interview data, it is important to recognize that this sample of seventeen was relatively small and represents the views of only one group. Examination of other existing groups like SVI could generalize these findings with more certainty. Additionally, because interpreters had to opt in and give their time, those who were willing to interview might have been particularly motivated and enthusiastic about interpreting.

It is also necessary to note that this is self-reported data from students who have not yet begun their formal medical education. As discussed previously, a student’s choices and thought processes while in school may influence their future career behavior post-training; for example, it has been shown that practicing community engagement prepares students to continue such work when they become physicians [22, 30]. But it is important to acknowledge that undergraduate activities do not dictate precisely how an individual’s career will pan out. On one hand, experiences as a student interpreter might have positive effects even beyond those that have been described here. On the other hand, it is certainly possible that experiences as interpreters might contribute to some students turning away from healthcare. Ultimately, exploring the effect that interpretation has on students past their undergraduate careers will be an important topic for future research to address.

Next, it is important to consider the effect that student interpretation has on the field of professional interpretation. Though student interpreters could easily take their skills and experience to careers in interpretation, it seems noteworthy that none of the SVI students in this study were planning to become professional interpreters, despite the very positive experiences they had with interpretation. This study did not probe further into the students’ thoughts on becoming full-time interpreters, but we should reflect on what it means for the interpreter workforce to have some people participating for a time as students and then pivoting toward other roles.

Finally, as much as we hope that student interpreters help patients, speaking only to the interpreters as we did in this study unfortunately does not include the voice of the patient. To ensure that patients really are getting the most linguistically appropriate care, it would be essential to understand the patient perspective on the work of student interpreters.

There are several challenges that come with interpreting: chiefly, the investment of both time and money in the training course, as well as the difficulty of the work itself. However, there are also numerous rewards, ranging from concrete improvement in language skills to more abstract practice of interpersonal skills. The intricate balance of difficulties, effort, and outcomes parallel some of the sacrifices and successes that trainees experience on the way to a career in healthcare. Purposefully examining student volunteers brings them to the forefront of the conversation on interpretation, which has so far only touched upon this demographic in a few cases. Exploring this balance provides valuable insight into how this extracurricular activity serves students interested in medical careers both personally and professionally. Finally, SVI students’ experiences have the direct application of encouraging the development of student interpreter programs at other universities, allowing more students to contribute both to their communities in the short term and their future patients in the long term.

### Electronic supplementary material

Below is the link to the electronic supplementary material.

Supplementary Material 1

Supplementary Material 2

## Data Availability

The corresponding author may be contacted for the complete codebook used in this study. Data sharing is not applicable to this article as no datasets were generated during the current study.

## Footnotes

1 To protect interviewees’ privacy, they are referred to by an assigned code (e.g. I1 or I2 to represent Interpreter 1, Interpreter 2, etc.). Filler words were removed from some of the excerpts for clarity.

## Declarations

### Authors’ information

J.W. is a current medical student at the University of Pennsylvania and a former Fulbright U.S. Student grantee to Peru. She completed this work while an undergraduate at Emory University. S.T. is Teaching Professor and director of the Linguistics Program at Emory University. Her work focuses on sociolinguistics, language attitudes, and health communication. She is the co-author of *Language and Linguistic Diversity in the US: An Introduction* and *Linguistic Planets of Belief: Mapping Language Attitudes in the American South*, both published by Routledge.
