# Supplementary material for: Motivations of undergraduate student medical interpreters: Exposure and experience
Source: BMC Med Educ. 2024 Apr 24;24:444. doi: 10.1186/s12909-024-05417-y (PMC11040973; doi:10.1186/s12909-024-05417-y)
Supplement: Supplementary file 1 — Supplementary Material 1 [file 12909_2024_5417_MOESM1_ESM.docx]

**Appendix**

**Interview Guide**

Major differences between the interviews for more and less experienced interpreters are noted.

- Verbal consent
- Demographic questions:
  - What year in school are you?
  - What is your major?
  - What are your career interests?
  - What is your level of proficiency/experience with Spanish or Portuguese?
  - If you feel comfortable sharing, where did you grow up? What is your ethnicity? How old are you? What is your gender?
- Interview questions:
  - When did you first get involved in SVI? How did you hear about it? What was the application and recruitment part like?
  - [More experienced]: Why did you choose to participate in SVI initially? Why do you continue to participate?

[Less experienced]: What made you want to be involved? How do you feel now that you are in SVI?

- - What do you (see as/predict will be) the most significant challenges you face while interpreting? What’s the most difficult thing to translate/interpret?
  - What do you (see as/predict will be) the greatest benefits to you as an interpreter? To the patients and doctors?
  - How do you see yourself in comparison to professional interpreters? Do you feel more/less familiar with the language and cultural knowledge? Do you feel that you can relate better/worse to some patients than a professional interpreter? Do the patients and doctors know that you are students? If so, does that come up in the interaction?
  - How do you keep up with the interpretation as they are talking?
  - Please describe your interactions with the other members of SVI.
  - What do you think are the most important qualities for someone to have to be an interpreter?
  - For more experienced only:
    - Describe a typical encounter with a patient and doctor.
    - Tell me about your most memorable SVI encounter.
    - What advice would you give to someone who is interested in becoming a student medical interpreter?
  - For less experienced only:
    - What do you see as the role of the interpreter?
    - How do you think your age will affect your interpreting?
    - Are there any downsides to interpreting?
    - What are you most looking forward to about starting to interpret? What are you most scared of when it comes to interpreting?
  - Anything else you’d like to share that we haven’t covered already or go back and expand on?
