# Supplementary material for: Motivations of undergraduate student medical interpreters: Exposure and experience
Source: BMC Med Educ. 2024 Apr 24;24:444. doi: 10.1186/s12909-024-05417-y (PMC11040973; doi:10.1186/s12909-024-05417-y)
Supplement: Supplementary file 2 — Supplementary Material 2 [file 12909_2024_5417_MOESM2_ESM.docx]

**Codebook**

Only select subcodes are presented for conciseness. Please contact Julie Wechsler ([julie.wechsler@pennmedicine.upenn.edu](mailto:julie.wechsler@pennmedicine.upenn.edu)) for a more detailed codebook if interested.

| Section | Parent Code | Subcodes |
| --- | --- | --- |
| Demographics | Year in School  Age  Gender  Ethnicity  Major  Career interests      Language background | Pre-health career track  Working as an interpreter  Other career (non-healthcare) |
| Interpretation | Benefits to the patients (or goals of the interpreter)    Benefits to the doctors    Challenges    Personal experience interpreting    Role of the interpreter        Language | Better communication with doctors    Learn to work with interpreters    Accuracy    Most memorable session    Professional interpreter  Student volunteer interpreters  Ad hoc interpreters    Dialectal differences |
| Motivations & Benefits | Motivation to start          Benefits to the interpreters | Language/culture/family  Health-related experience  Contribution to community  Multifactor motivation    Professional experience  Personal gratification  Direct impact |
| SVI-specific | Intro to SVI    Training      Interactions as a SVI member      Barriers to interpreting | Where did you hear about it?    Training description  What they learn    Feelings about SVI  Interactions with members    Time cost of interpreting  Financial cost of training |
